# Variability in biopsy quality informs translational research applications in hepatocellular carcinoma

**DOI:** 10.1038/s41598-021-02093-6

**Published:** 2021-11-23

**Authors:** Kelley Weinfurtner, Joshua Cho, Daniel Ackerman, James X. Chen, Abashai Woodard, Wuyan Li, David Ostrowski, Michael C. Soulen, Mandeep Dagli, Susan Shamimi-Noori, Jeffrey Mondschein, Deepak Sudheendra, S. William Stavropoulos, Shilpa Reddy, Jonas Redmond, Tamim Khaddash, Darshana Jhala, Evan S. Siegelman, Emma E. Furth, Stephen J. Hunt, Gregory J. Nadolski, David E. Kaplan, Terence P. F. Gade

**Affiliations:** 1 Division of Gastroenterology and Hepatology, University of Pennsylvania, Philadelphia, PA USA; 2 Penn Image-Guided Interventions Laboratory, University of Pennsylvania, Philadelphia, PA USA; 3 Department of Radiology, University of Pennsylvania, Philadelphia, PA USA; 4 Vascular & Interventional Specialists of Charlotte Radiology, Charlotte, NC USA; 5 Perelman School of Medicine, University of Pennsylvania, Philadelphia, PA USA; 6 Division of Interventional Radiology, University of Pennsylvania, Philadelphia, PA USA; 7 Corporal Michael J Cresenz VA Medical Center, Philadelphia, PA USA; 8 Department of Pathology and Laboratory Medicine, University of Pennsylvania, Philadelphia, PA USA; 9 Radiology and Cancer Biology, University of Pennsylvania Perelman School of Medicine, 652 BRB II/III, 421 Curie Blvd, Philadelphia, PA 19104-6160 USA

**Keywords:** Liver cancer, Hepatocellular carcinoma, Tumour heterogeneity, Translational research

## Abstract

In the era of precision medicine, biopsies are playing an increasingly central role in cancer research and treatment paradigms; however, patient outcomes and analyses of biopsy quality, as well as impact on downstream clinical and research applications, remain underreported. Herein, we report biopsy safety and quality outcomes for percutaneous core biopsies of hepatocellular carcinoma (HCC) performed as part of a prospective clinical trial. Patients with a clinical diagnosis of HCC were enrolled in a prospective cohort study for the genetic, proteomic, and metabolomic profiling of HCC at two academic medical centers from April 2016 to July 2020. Under image guidance, 18G core biopsies were obtained using coaxial technique at the time of locoregional therapy. The primary outcome was biopsy quality, defined as tumor fraction in the core biopsy. 56 HCC lesions from 50 patients underwent 60 biopsy events with a median of 8 core biopsies per procedure (interquartile range, IQR, 7–10). Malignancy was identified in 45/56 (80.4%, 4 without pathology) biopsy events, including HCC (40/56, 71.4%) and cholangiocarcinoma (CCA) or combined HCC-CCA (5/56, 8.9%). Biopsy quality was highly variable with a median of 40% tumor in each biopsy core (IQR 10–75). Only 43/56 (76.8%) and 23/56 (41.1%) samples met quality thresholds for genomic or metabolomic/proteomic profiling, respectively, requiring expansion of the clinical trial. Overall and major complication rates were 5/60 (8.3%) and 3/60 (5.0%), respectively. Despite uniform biopsy protocol, biopsy quality varied widely with up to 59% of samples to be inadequate for intended purpose. This finding has important consequences for clinical trial design and highlights the need for quality control prior to applications in which the presence of benign cell types may substantially alter findings.

## Introduction

The development of precision medicine has resulted in a dramatic increase in image-guided percutaneous biopsies performed for research indications^1–5^. This increase has raised awareness regarding the responsibilities incumbent on physicians acquiring these biopsies, specifically, the importance of providing an accurate assessment of individual risk as well as the likelihood of acquiring samples that can be utilized for the intended clinical and research applications^3,4^. While the diagnostic accuracy and complication rates of percutaneous biopsies for clinical purposes have been documented, research biopsy protocols often require larger volumes of tissue and are used for multiple research endpoints, such as histologic, genomic, transcriptomic, and immune profiling^6,7^. In this context, the complication rates may differ from clinical biopsies, and metrics for biopsy quality are likely to be assay dependent with pathologic diagnosis being only one measure. Inadequate biopsy quality may lead to repeat procedures and increase in exposure to complications, the requirement for larger sample sizes in clinical trials, and/or unrepresentative or misleading data. Oncology clinical trials have not consistently reported biopsy protocols and outcomes, and, as such, the majority of the existing data is derived from retrospective analyses of variable biopsy methods and metrics of biopsy adequacy^8–18^. Moreover, these studies have largely been limited to adequacy for nucleic acid and histologic applications. As a result, there is limited data regarding biopsy quality for research applications in which the amount of non-tumor tissue may impact the results, including proteomic and metabolomic profiling and the development of patient-derived models.

Hepatocellular carcinoma (HCC) is a unique example of a malignancy for which biopsies have played a limited clinical role, but research biopsies are becoming an increasingly central part of characterizing tumor biology and facilitating the development of targeted therapies for personalized medicine approaches. We aimed to investigate the safety of percutaneous core biopsies of HCCs acquired immediately prior to locoregional therapy (LRT) as well as the quality of these biopsies and its impact on downstream clinical and research applications.

## Materials and methods

### Enrollment

**Figure 1:**
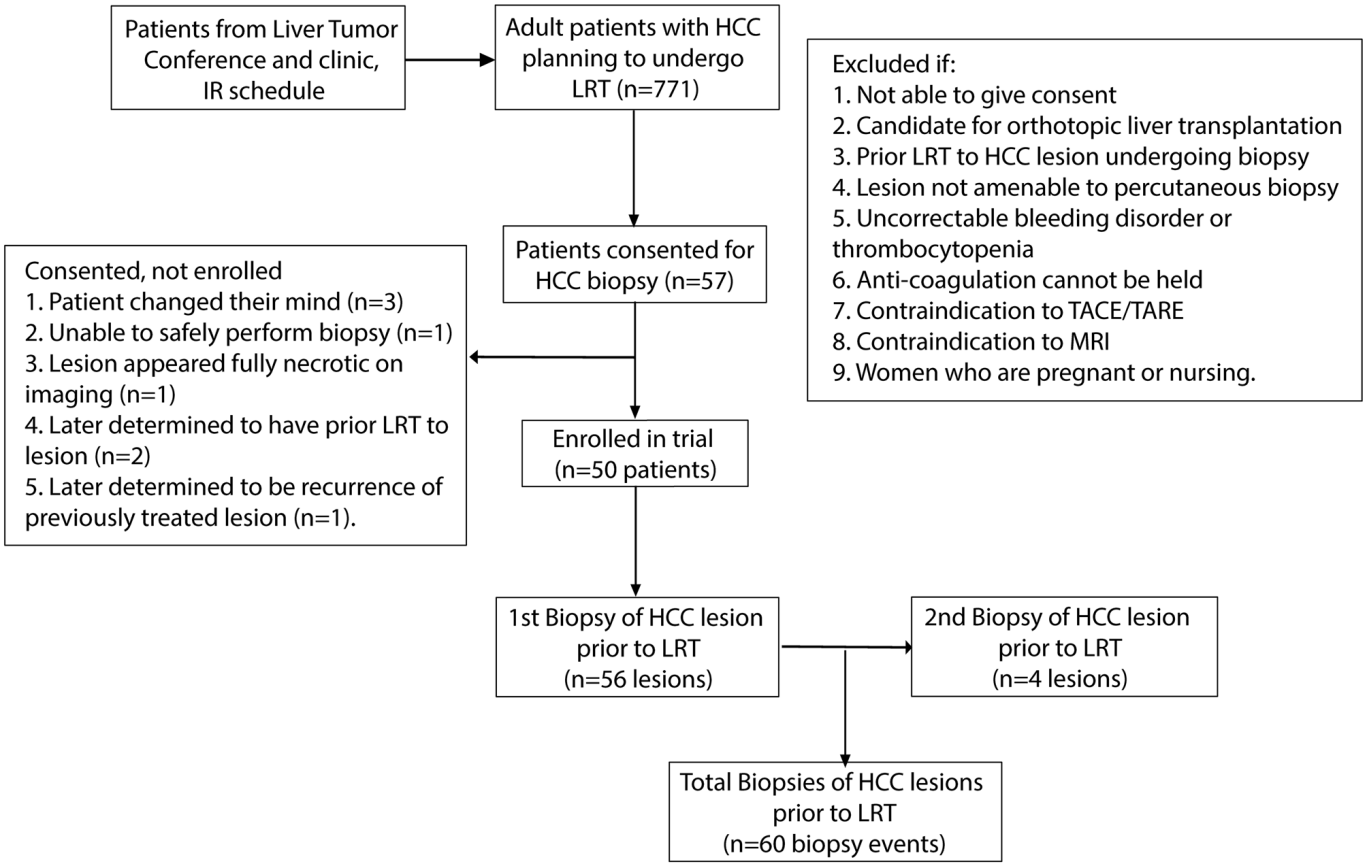
Patient Enrollment. Schematic demonstrating patient enrollment in the prospective clinical trial.

Patients were recruited from the Interventional Oncology Clinic and multidisciplinary Hepatic Tumor Clinics at two tertiary care academic institutions for enrollment into a prospective cohort study for the genetic, proteomic, and metabolomic profiling of HCC and the development of patient-derived models (Fig. 1). This study was approved by Institutional Review Boards (IRB) at both institutions (University of Pennsylvania IRB #823696 and Corporal Michael J Crescenz VA Medical Center IRB #01779) and all research was performed in accordance with their guidelines/regulations, including obtaining informed consent from all participants. Patients were eligible if they (1) were ≥ 18 years old; (2) were capable of giving informed consent; (3) had a clinical diagnosis of HCC; and (4) planned to undergo LRT. Patients were excluded if they (1) were a candidate for orthotopic liver transplantation; (2) had prior LRT to the target HCC lesion; (3) had a target HCC that was not amenable to computed topography (CT) or ultrasound (US)-guided percutaneous biopsy as determined by the treating interventional radiologist; (4) had an uncorrectable thrombocytopenia/bleeding disorder or anti-coagulation could not be held; or (5) had a contraindication to contrast-enhanced magnetic resonance imaging (MRI) for follow-up imaging.

### Biopsy procedure

**Figure 2:**
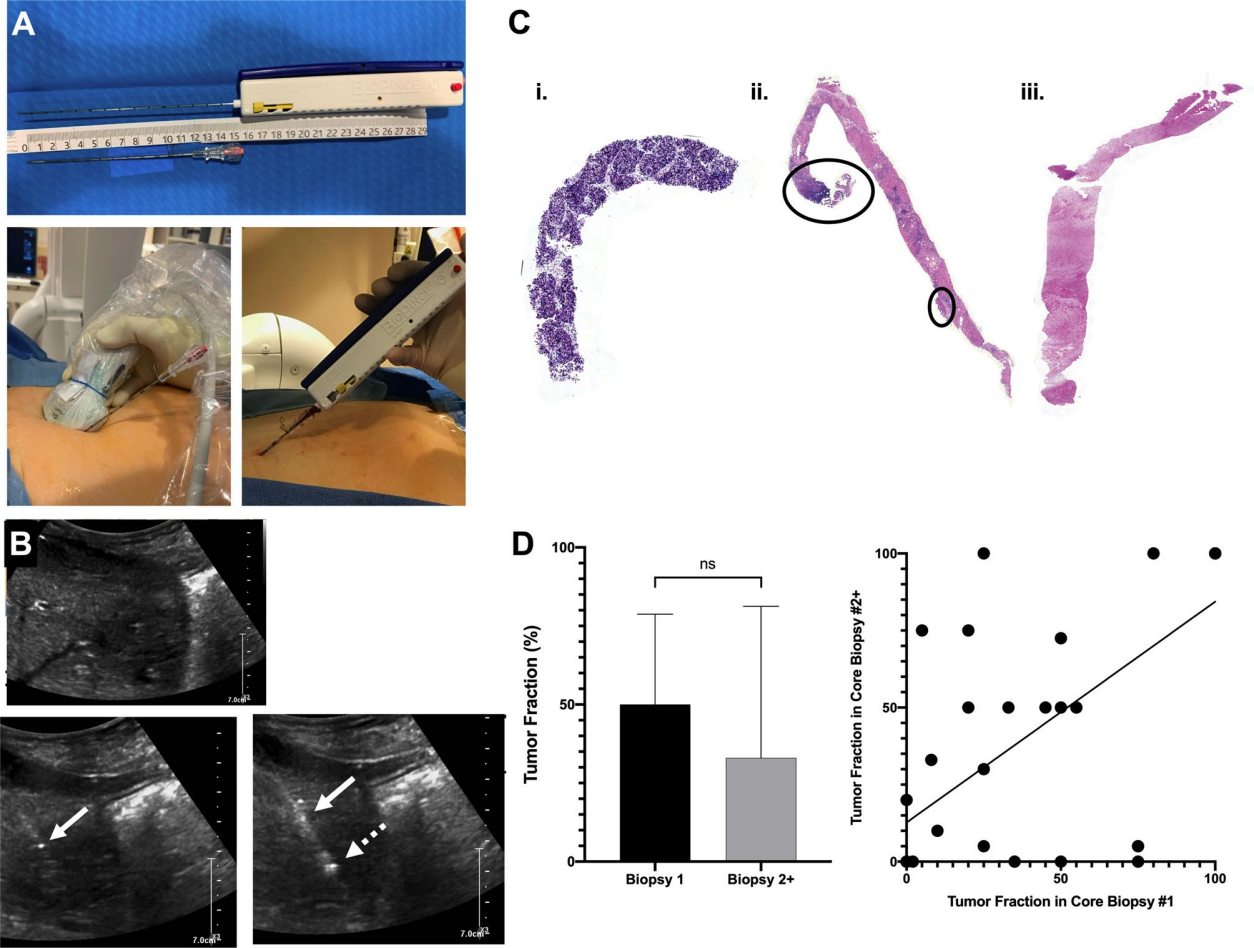
Percutaneous liver biopsy technique and quality. (**A**) Representative images of 17G coaxial trocar and 18G BioPince core biopsy needle. (**B**) Representative US images demonstrating segment 2 tumor with intra-tumoral positioning of the trocar (solid arrow) and coaxial deployment of the core biopsy needle (dashed arrow). (**C**) Representative images of core biopsies demonstrating heterogeneity of tumor content: (i) 100% tumor; (ii) 10% tumor; (iii) no tumor. (**D**) Bar graph comparing the average tumor fraction in the first biopsy and subsequent biopsy cores (p = 0.75). Scatter plot demonstrating correlation of tumor fraction in first and subsequent biopsy cores (r = 0.66, R^2^ = 0.43, p < 0.0001).

All biopsies were performed by attending interventional radiologists. Briefly, the patient was positioned in the supine position and moderate sedation was administered. For patients undergoing endovascular LRT, the catheter was positioned into the target vessel and pre-embolization arteriography was performed. Subsequently, the target lesion was identified on US and/or CT. Under imaging guidance, a 17G trocar needle was advanced through the tumor capsule (Fig. 2A + B). An 18G BioPince Full Core biopsy needle (Argon Medical, Frisco, TX) was inserted coaxially and used to obtain core biopsies of adequate quality based on tissue volume and integrity as determined by the operator. The trocar reoriented in several positions during the course of procedure in order to sample diverse tumor regions. After removal of the biopsy needle, hemostasis was achieved through manual compression and/or tract embolization using GelFoam Compressed Sponge suspension at the discretion of the operator. Planned LRT was then performed using standard protocols.

### Sample processing

At the discretion of the operator, up to four core biopsy samples were sent directly to Surgical Pathology for standard clinical care processing, including formalin fixation and paraffin-embedding with subsequent hematoxylin and eosin staining for histology review. The remaining core biopsies were snap frozen for genomic, proteomic, and/or metabolomic profiling or incubated in cell culture medium on ice for the generation of patient-derived models including cell lines and/or patient-derived xenografts (PDX) as previously described^19^.

### Data collection

All study data was prospectively collected and managed using a Research Electronic Data Capture database. All follow-up cross-sectional imaging was reviewed by a single fellowship-trained body radiologist (ES) with more than 25 years of experience to evaluate for complications with a minimum follow-up of one MRI performed 30 days after procedure. Histology slides were reviewed by hepatobiliary pathologists (EEF or DJ) with more than 25 years of experience for the presence of malignancy, grade of differentiation, and tumor fraction of core biopsy. Allele frequencies of telomerase reverse transcriptase (TERT) promoter mutations were determined by droplet digital polymerized chain reaction, while allele frequencies for CTNNB1 and TP53 mutations were determined by a custom next generation sequencing panel. Tumor fraction cutoffs for downstream research applications were determined by established laboratory protocols from the Penn Center for Personalized Diagnostics, Penn Quantitative Proteomics Resource Core, and Metabolon Inc (Durham, NC).

Patient demographics, disease-specific information, and procedure details were obtained from chart review and discussion with the treating interventional radiologist. The size of the lesion was defined by largest diameter on most recent cross-sectional imaging and the location of the lesion was defined by (i) segment occupied, considered “multiple” if spanning more than one segment, (ii) lobe occupied, (iii) region occupied using posterior/superior (segments 1, 4A, 7, 8) and anterior/inferior (segments 2, 3, 4B, 5, 6). Of 12 total operators, only 4 performed more than 5 biopsies and were defined as operators 1–4 with all the other operators combined into “other.”

### Outcomes and statistical analysis

The primary outcome was biopsy quality defined as the average tumor fraction of all the core biopsies sent for histologic evaluation for that biopsy event (average 2.2 core biopsies per event, range 0–5). The secondary outcomes were (1) overall and major complication rates with major complication defined as grade 3 or above (i.e. required blood transfusions, invasive intervention, and/or hospitalization), (2) biopsy adequacy for downstream research applications as defined by tumor fraction ≥ 10% for genomic and ≥ 50% proteomic/metabolomic profiling, (3) percentage of biopsy events that resulted in the establishment of PDX model, and (4) PDX engraftment rate defined as the number of first-generation HCC PDXs established divided by the total number of mice implanted with tissue from one biopsy event. Clinical characteristics and biopsy outcomes were compared using Pearson’s chi square, Fisher’s exact, Student’s t-test, Wilcoxon rank-sum, Kruskal–Wallis tests, or Pearson pairwise correlation. Multivariable models were analyzed by logistic and linear regression. PDX engraftment rates were compared to biopsy quality and mutation allele frequency using linear regression. All data were analyzed using Stata/IC 16.1.

## Results

### Patient and biopsy characteristics

From April 2016 to July 2020, charts from 771 patients diagnosed with HCC and referred for LRT were evaluated with 64 patients eligible for enrollment. 57 patients were consented with 50 patients ultimately enrolled for a total of 60 biopsy events (Fig. 1). Patient characteristics at the time of biopsy are summarized in Table 1. Most patients had underlying cirrhosis (53/60, 88.3%), but were well-compensated at the time of biopsy (49/60 Child Pugh A, 81.7%) with a median model for end-stage liver disease (MELD) score of 8 (IQR 7–10). Of the five patients without cirrhosis, one had non-alcoholic fatty liver disease and the other 4 had no evidence of cirrhosis nor clear risk factors for HCC (4 patients accounting for 6 biopsy events, 6/60, 10.0%). The majority of patients had Barcelona clinic liver cancer (BCLC) stage B or C disease (26/60 stage B, 43.3% and 23/60 stage C, 38.3%, respectively) with median size of the target lesion of 4.2 cm (IQR 2.7–8.5) and median of 8 core biopsies taken per procedure (IQR 7–10) for a total of 499 core biopsies.

**Table 1:** Patient and biopsy characteristics.

	Biopsy events (n = 60)
**Age (SD)**	65.0 (8.3)
**Gender (%)**	
Male	51 (85.0)
Female	9 (15.0)
**Race/ethnicity (%)**	
White	42 (70.0)
Black/African American	13 (21.7)
Hispanic/Latino	3 (3.3)
Asian	2 (5.0)
**Etiology of HCC (%)**	
HCV/alcohol	20 (33.3)
HCV	14 (23.3)
Alcohol	8 (13.3)
NAFLD	6 (10.0)
HBV	2 (3.3)
Other	4 (6.7)
Unknown	6 (10.0)
**Underlying cirrhosis (%)**	53 (88.3)
**MELD score (IQR)**	8 (7–10)
**Childs-Pugh score (%)**	
A	49 (81.7)
B	11 (18.3)
C	0
**BCLC stage (%)**	
A	11 (18.3)
B	26 (43.3)
C	23 (38.3)
**AFP (ng/mL, IQR)**	14 (6–216)
**Size of lesion (cm, IQR)**	4.2 (2.7–8.5)
**Location of lesion**	
Right lobe (%)	42 (70.0)
Posterior/superior (%)	16 (29.6)
Spans multiple segments (%)	20 (33.9)
**Distance of lesion from capsule (cm, IQR)**	3.5 (1.5–5.5)
**Number of core biopsies (IQR)**	8 (7–10)
**Gelfoam used (%)**	42 (70.0)
**Imaging guidance (%)**	
US only	53 (88.3)
CT only	6 (10.0)
US and cone beam CT	1 (1.7)

### Biopsy outcomes

Malignancy was diagnosed in 45/56 lesions (80.3%, 4 not sent for surgical pathology) with diagnoses of HCC in 40/56 (71.4%) and cholangiocarcinoma (CCA) or combined HCC-CCA (cHCC-CCA) in 5/56 (8.9%). Based on follow-up imaging, alpha-fetoprotein (AFP), and clinical course, the overall diagnostic accuracy was 47/56 (83.9%) with 9/56 (16.1%) false negatives-noting that only two of these nine lesions were subsequently confirmed as HCC by biopsy. On univariable analyses of clinical characteristics, the only factor associated with the likelihood of a diagnostic biopsy was the etiology of HCC (90% in hepatitis C virus/alcohol, 83.3% in alcohol, 64.3% in hepatitis C virus, 50% in non-alcoholic fatty liver disease, 100% in other, p = 0.04, Supplementary Table 1). There was no significant association between diagnostic biopsy and patient demographics, presence of underlying cirrhosis, BCLC stage, MELD, size of lesion, location of lesion by region or lobe, distance of lesion from the liver capsule, operator, or imaging guidance. On multivariable analysis that included the etiology of HCC, location of lesion by region, and distance of lesion from capsule, no factors were associated with the likelihood of a diagnostic biopsy.

Biopsy quality varied widely with a median tumor fraction of 40% (IQR 10–75%). Representative biopsy cores are illustrated in Fig. 2C. Notably, the quality of the first core biopsy correlated with the biopsy quality of subsequent core biopsies acquired in the same encounter (r = 0.66, R^2^ = 0.43, p < 0.0001, Fig. 2D). On univariable analyses of clinical characteristics, biopsy quality was not associated with patient demographics, etiology of HCC, presence of cirrhosis, BCLC stage, MELD, size of lesion, location of lesion, or type of imaging guidance (Table 2). Biopsy quality differed between HCC subtypes with median percent tumor of 75% for clear cell type compared with 20% for steatohepatitis-associated; however, this was not statistically significant due to small sample sizes in these subtypes. Biopsy quality also varied by operator (p = 0.02) and the number of core biopsies sent for pathologist review (p = 0.02); however, on multivariable analysis that included gender, MELD score, operator, image guidance, and number of biopsy cores sent for pathology, these associations were no longer significant. While all patients in our study carried a clinical diagnosis of HCC and had been referred for LRT, on review of pre-procedure imaging for liver reporting and data system (LIRADS) score, the lesions were characterized as 44/60 (73.3%) LIRADS 5, 9/60 (15.0%) LIRADS TIV, 6/60 (10.0%) LIRADS 4, and 3/60 (5.0%) LIRADS M. Notably, LIRADS score was not associated with diagnostic biopsy or biopsy quality.

**Table 2:** Factors associated with biopsy quality.

	Median tumor fraction (IQR) or correlation coefficient	p-value	Multiple linear regression coefficient (95% CI)	p-value
**Age**	− 0.09	0.51		
**Gender**		0.06		0.10
Male	100 (40–100)		(ref)	
Female	35 (10–63)		20.54 (− 4.28 to 45.36)	
**Race/ethnicity**		0.34		
White	34 (10–63)			
Black/African American	65 (18–100)			
Hispanic/Latino	40 (0–42)			
Asian	70 (40–100)			
**Etiology of HCC**		0.33		
HCV/alcohol	34 (13–73)			
HCV	38 (0–75)			
Alcohol	54 (10–80)			
NAFLD	20 (0–40)			
Other	56 (40–100)			
**MELD score**	0.25	0.06	2.20 (− 1.36 to 5.76)	0.22
**Underlying cirrhosis**		0.24		
Yes	36 (10–75)			
No	63 (40–100)			
**BCLC stage**		0.48		
A	40 (0–50)			
B	48 (15–90)			
C	33 (10–75)			
**AFP (ng/mL, IQR)**	0.04	0.80		
**Size of lesion (cm, IQR)**	− 0.07	0.62		
**Location of lesion**				
Multiple vs single segment(s) (%)	43 (10–80) vs 40 (5–65)	0.75		
Post/Sup vs Ant/Inf (%)	45 (13–78) vs 36 (5–75)	0.54		
Right vs left lobe (%)	40 (10–75) vs 40 (10–65)	0.81		
Distance of lesion from capsule (cm)	− 0.18	0.20		
**Operator**		0.02*		
1	83 (50–100)		8.42 (− 21.47 to 38.30)	0.57
2	30 (0–42)		− 9.15 (− 33.21 to 14.92)	0.45
3	63 (42–75)		18.75 (− 20.87 to 58.36)	0.35
4	10 (5–10)		− 26.87 (− 64.16 to 10.42)	0.15
Other	50 (21–90)		(ref)	
**Imaging guidance**		0.13		
US only	40 (10–80)		(ref)	0.21
CT used	28 (0–42)		18.27 (− 47.34 to 10.80)	
Number of biopsies sent for pathology	0.31	0.02*	8.11 (− 3.67 to 19.90)	0.17

**Figure 3:**
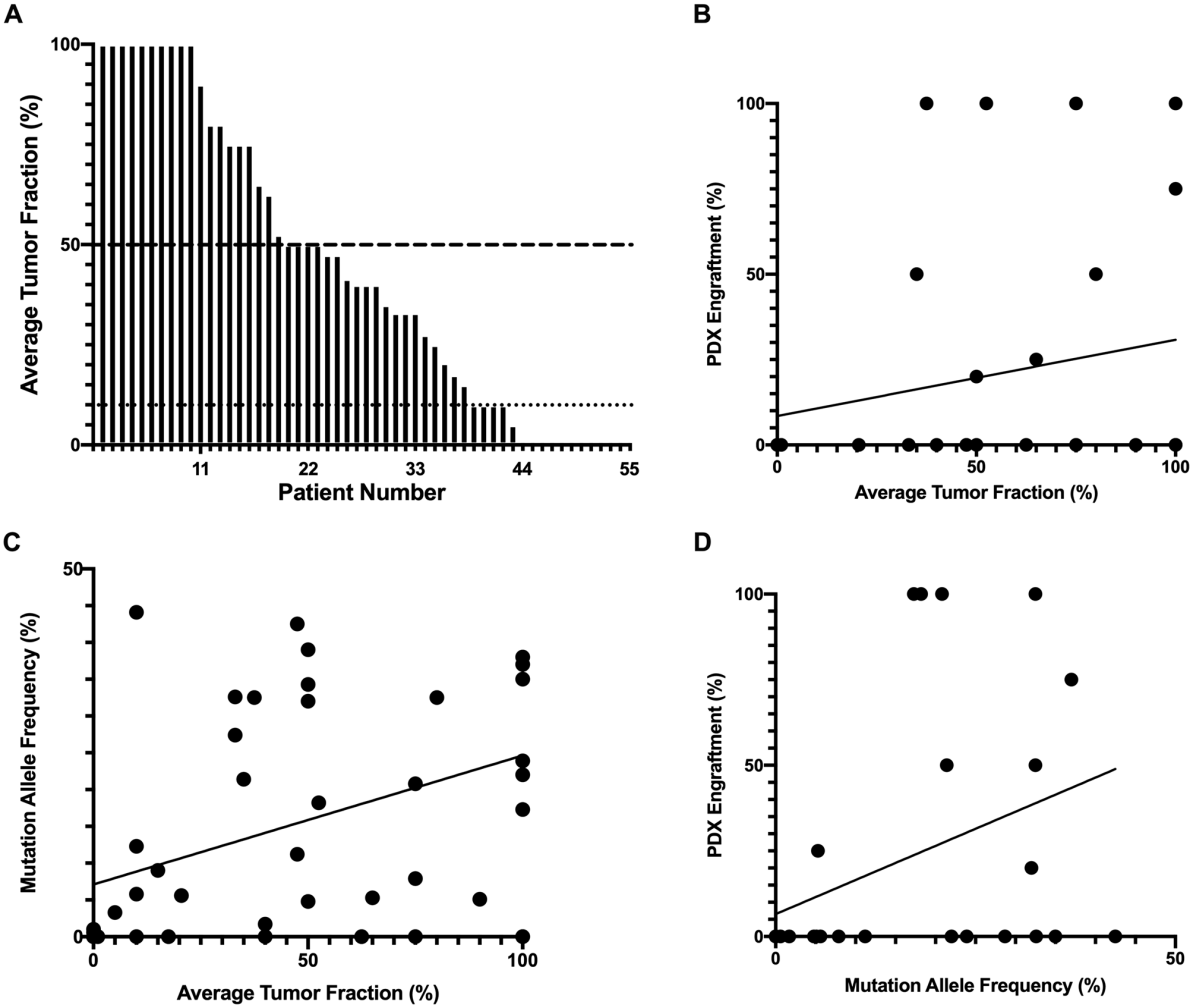
Biopsy adequacy for downstream applications. (**A**) Bar graph showing average tumor fraction by biopsy event with cutoffs for next generation sequencing (dotted line) as well as proteomic and metabolomic profiling (dashed line). (**B**) Scatter plot of average tumor fraction in biopsies and percentage of patient-derived xenografts (PDXs) engrafting for individual patients (r = 0.22, R^2^ = 0.05, p = 0.24). (**C**) Scatter plot of average tumor fraction in biopsies and mutation allele frequency from same biopsy event (r = 0.39, R^2^ = 0.14 = 5, p = 0.008). (**D**) Scatter plot of mutation allele frequency and percentage of PDXs engrafting for individual patients (r = 0.38, R^2^ = 0.14, p = 0.048).

Based on assay-specific cutoffs for tumor fraction, biopsies were adequate for next generation sequencing (tumor fraction ≥ 10%) in 43/56 (76.8%) of cases and for proteomic/metabolomic profiling (tumor fraction ≥ 50%) in 23/56 (41.1%) cases (Fig. 3A). Biopsy quality did not correlate with the successful generation of PDXs (58.8% vs 47.5%, p = 0.42) or the PDX engraftment rate (r = 0.22, R^2^ = 0.05, p = 0.24, Fig. 3B); however, it did correlate with the allele frequency of pathogenic mutations (r = 0.39, R^2^ = 0.14 = 5, p = 0.008, Fig. 3C) and the allele frequency correlated with an increased PDX engraftment rate (r = 0.38, R^2^ = 0.14, p = 0.048, Fig. 3D).

The overall and major complication rates were 5/60 (8.3%) and 3/60 (5.0%), respectively, with 2 major bleeding events (2/60, 3.3%), 2 minor bleeding events (2/60, 3.3%), and 1 potential biopsy tract seeding event (1/60, 1.7%). Regarding the two major bleeding events, one was intra-abdominal and the other was a hemothorax due to injury to an intercostal artery, both of which required drainage by interventional radiology. Regarding the two minor bleeding events, one patient presented to the Emergency Room 9 days post-procedure with right flank pain and was found to have a subacute retroperitoneal bleed which was observed without intervention or admission. The second patient had an asymptomatic intra-hepatic hematoma found on follow-up imaging. No patients required blood transfusions for bleeding. Finally, the single case of biopsy tract seeding was suspected based on new peritoneal implants and enhancement along the biopsy tract seen on the first follow-up imaging 125 days post-procedure in a patient ultimately diagnosed with CCA. This seeding was not confirmed by tissue sampling or follow-up imaging as the patient passed away within the next month. Complications were associated with female gender (33.3% vs 4.2%, p = 0.03) and non-white race/ethnicity (23.1% in Black/African American, 20.0% in other vs 2.4% in white/Caucasian, p = 0.03) (Supplementary Table 2). More complications were observed in patients with CCA or cHCC-CCA (40.0% vs 7.5% in HCC, 0% in no malignancy, p = 0.07). Sample size was too small to allow for multivariable analysis.

## Discussion

This study reports quality and safety outcomes of percutaneous core biopsies of HCC acquired in the context of a prospective clinical research study. Biopsy quality varied widely with median tumor fraction < 50% and 23–59% of biopsies insufficient for research applications depending on the assay. The unanticipated inadequacy of biopsy samples delayed the achievement of the primary study outcomes, requiring an expansion from 50 to 100 patients. There were no clear patient, tumor, or biopsy characteristics associated with diagnostic biopsy or biopsy quality on multivariable analysis, though there was trend toward a lower proportion of diagnostic biopsies and biopsy quality in patients with non-alcoholic fatty liver disease (NAFLD). This finding may be related to previously reported challenges with distinguishing HCCs in the context of the larger body habitus and/or hepatic steatosis associated with NAFLD^20^. The lack of factors predictive of biopsy quality combined with competing dispositions for the acquired tissue highlights the importance of non-destructive, point-of-care assessments in determining biopsy adequacy for diagnostic purposes and downstream applications. Finally, despite the large number of core biopsies acquired per patient, clinically significant complications were encountered in only 5% of patients.

While evaluations of diagnostic biopsies rely on qualitative histologic assessments, the adequacy of biopsy samples for molecular testing is determined by quantitative assessments of tumor fraction with discrete thresholds applied based on assay-specific features. The heterogeneity in tumor fraction identified in this study was consistent with findings in prior studies^13,17^, as was the adequacy of biopsies for nucleic acid or slide-based assays (77% vs 57–93% in prior studies)^11–13,15,17,18,21–25^. While sequencing analyses utilize variant calling to distinguish pathologic mutations from noise, other forms of molecular testing, including metabolomic and proteomic profiling, lack specificity in distinguishing signatures of malignant versus benign cell types. As a result, the presence of benign cell types may substantially alter the findings, such that current standards emphasize the use of tissue samples in which the target malignant cell type constitutes at least 50% of the sample. Based on this threshold, only 41% of biopsies were adequate for metabolomic or proteomic profiling, a primary objective of our study. The influence of biopsy quality on other downstream applications, such as patient-derived models, has not been established. In the current study, while tumor fraction did not correlate with the likelihood of PDX establishment, the allele frequency of pathogenic mutations was associated with increased rates of PDX engraftment, suggesting that molecular analyses may provide an additional, and potentially more accurate, index of biopsy quality. Taken together, these data emphasize the unmet need to characterize and optimize the quality of research biopsies prior to use in research applications.

A primary obstacle in determining biopsy quality for downstream applications has been the destructive nature of current assessment methods. Interestingly, our study showed that the tumor fraction in the initial core biopsy submitted for pathologic diagnosis correlated with the tumor fraction of core biopsies acquired subsequently (median absolute difference 5%, IQR 0–30%), suggesting the potential for analyzing only a representative sentinel core. However, this approach requires delays associated with histologic processing and analysis, which may not be feasible for all assays. Several groups have proposed potential strategies to circumvent this problem. Dowlati et al. reported snap freezing biopsies and then dissecting the cores on dry ice, ensuring any tissue used for biochemical analysis is flanked by tissue sent for histology to confirm tumor, though this approach is limited to applications requiring small amounts of fresh frozen tissue and may be confounded by heterogeneity of tissue along the length of the biopsy as shown in Fig. 2C ^23^. Fine needle aspiration with cell block has been shown to have higher tumor fraction than core biopsies (67 vs 36%, respectively) with significantly less stromal components and benign hepatocytes^17^. This increased tumor fraction allowed for an 18% increase in sample adequacy for next generation sequencing (57% increased to 75%) though at the expense of preserving tissue architecture and the tumor microenvironment. In addition, rapid onsite pathology evaluation performed on cell aspirates derived from fine needle aspiration or on cytology imprint for core biopsies has been reported to have a positive predictive value for malignancy. However, this approach requires appropriate expertise and resources, results are generally qualitative not quantitative, and it is limited by the inability to distinguish hepatocellular adenomas, regenerative nodules, and well-differentiated HCC^17,26,27^. Alternative imaging techniques may allow for better target localization, including avoiding areas of necrosis and/or fibrosis. We found no difference in biopsy quality when using US alone, CT alone, or a combination of US and CT, and, while the number of CT-guided biopsies was small, this finding is consistent with prior data^28^. Contrast-enhanced US, however, may increase diagnostic yield and tumor fraction in percutaneous biopsies and techniques are being developed to overcome the technical challenges of MRI-guided biopsies in other solid tumors^13^. While flurodeoxyglucose (FDG) positron emission tomography (PET)-CT has been demonstrated to improve biopsy yield in certain tumors, its utility for HCC is more limited given the established variability in FDG avidity^29–31^. Lastly, liquid biopsy techniques have been promising in their ability to avoid complications associated with biopsies and potentially better capture the intra- and inter-tumor heterogeneity seen in HCC; however, these techniques have several limitations, including the necessity for special preparation of samples with highly specialized laboratory equipment; low levels of detection in early stage disease; and, perhaps most importantly, the inability capture the complexities of the tumor structure and microenvironment, which has proven to be important in predicting response to immunotherapy in HCC^32,33^. Ultimately, innovative imaging techniques and a rapid, non-destructive, point-of-care test of biopsy quality would be of great utility in optimizing both diagnostic and research biopsies.

Importantly, the reported data demonstrate that percutaneous core biopsies of HCC lesions acquired in the context of LRT have an acceptable safety profile with a major complication rate of 3/60 (5.0%) including 2/60 (3.3%) major bleeding events and 1/60 (1.7%) case of biopsy tract seeding. The incidence of seeding is below the reported median of 2.7% likely due to coaxial technique which limits number of passages through the liver capsule. While the major bleeding rate was slightly higher than previously reported, it is still under the Society of Interventional Radiology suggested quality improvement threshold of 5%^6,34^. Notably, the number of core biopsies taken was not associated with the rate of complications, consistent with prior studies using the coaxial technique^12,35^. Supporting this further, one of the two major bleeding events occurred from an intercostal artery during the placement of the trocar. Notably, despite all patients carrying a clinical diagnosis and undergoing LRT, biopsy revealed CCA or cHCC-CCA in 5/56 (8.9%), which would have changed clinical management in favor of trans-arterial radioembolization, external radiation, and/or systemic chemotherapy. Importantly, 2/3 (66.7%) of the major biopsy complications, including the only case of biopsy tract seeding, occurred in patients with CCA or cHCC-CCA, such that the highest complication rates occurred in patients whose clinical management was most informed by the biopsy results.

Designed and implemented by interventional radiologists, this study intentionally included features that enabled the assessment of biopsy quality and patient outcomes. First, the prospective design allowed the standardization of the biopsy technique and instruments. Prior studies, especially those examining biopsy of primary liver lesions, have mainly included retrospective study designs and often extended over time frames that limited standardization^8–18^. Second, the study design enabled prospective and comprehensive imaging follow-up, enhancing the sensitivity for the detection of complications. Indeed, one of the five bleeding events and the lone seeding event in our study were without clinical consequence, manifesting only as incidental findings on follow-up imaging. Finally, only patients with a clinical diagnosis of HCC were enrolled in this study, reducing confounding variables introduced by disparate patient populations and maximizing the likelihood of target tissue acquisition. The primary limitation of our study was the small sample size. The study population was primarily white and male with high fraction of hepatitis C viral infection and alcohol use that may limit generalizability in certain populations; however, this is consistent with the current HCC population in the United States. Finally, the targetability of individual lesions, and therefore suitability of each patient for enrollment, was left to the discretion of the patient’s interventional radiologist resulting in a potential source of confounding.

## Conclusions

In the era of precision medicine, biopsies play an essential role in the characterization of tumor biology, development of new therapies, and treatment paradigms, especially in cancers like HCC for which therapeutic options remain limited and tumor heterogeneity is a major issue. This study demonstrates that percutaneous core biopsies of HCC provide a rich resource for research applications and clinically actionable pathology data with an acceptable safety profile in the context of LRT. However, despite the relatively high diagnostic yield, the high frequency of cHCC-CCA or CCA in patients and the significant variability in biopsy quality has important implications for clinical trial design and emphasizes the need for better quality control measures prior to use of biopsy samples in downstream applications, especially those in which the presence of benign cell types may substantially alter the findings. Finally, no patient, tumor, or biopsy characteristics were predictive of biopsy quality, highlighting the need for a point-of-care based assay to detect presence and amount of tumor tissue.

## Supplementary Information

Supplementary Tables.

## Abbreviations

AFP: Alpha-fetoprotein
BCLC stage: Barcelona clinic liver cancer stage
CT: Computed topography
CCA: Cholangiocarcinoma
HCC: Hepatocellular carcinoma
cHCC-CCA: Combined hepatocellular and cholangiocarcinoma
FDG PET: Fluorodeoxyglucose positron emission tomography
HCV: Hepatitis C virus
HBV: Hepatitis B virus
IRB: Institutional review board
IQR: Interquartile range
LIRADS score: Liver reporting and data system score
LRT: Locoregional therapy
MELD: Model for end-stage liver disease
MRI: Magnetic resonance imaging
NAFLD: Non-alcoholic fatty liver disease
PDX: Patient-derived xenografts
SD: Standard deviation
TERT: Telomerase reverse transcriptase
US: Ultrasound
