# Supplementary material for: Variability in biopsy quality informs translational research applications in hepatocellular carcinoma
Source: Sci Rep. 2021 Nov 23;11:22763. doi: 10.1038/s41598-021-02093-6 (PMC8611010; doi:10.1038/s41598-021-02093-6)
Supplement: Supplementary file 1 — Supplementary Tables. [file 41598_2021_2093_MOESM1_ESM.docx]

| **Supplementary Table 1. Factors Associated with Diagnostic Biopsy** | | | | | |
| --- | --- | --- | --- | --- | --- |
|  | **Diagnostic Biopsy**  **(n=45)** | **No Malignancy**  **(n=11)** | **p-value** | **Multivariable**  **OR (95%CI)** | **p-value** |
| **Age (SD)** | 64.7 (7.2) | 64.9 (9.2) | 0.95 |  |  |
| **Gender (%)**  Female  Male | 7 (77.8)  38 (80.9) | 2 (22.2)  9 (19.1) | 1.0 |  |  |
| **Race/Ethnicity (%)**  White  Black/African American  Other | 30 (78.9)  11 (84.6)  4 (80.0) | 8 (21.1)  2 (15.4)  1 (20.0) | 1.0 |  |  |
| **Etiology of HCC (%)**  HCV  Alcohol  HCV/alcohol  NAFLD  Other | 9 (64.3)  5 (83.3)  18 (90.0)  3 (50.0)  10 (100.0) | 5 (35.7)  1 (20.0)  2 (10.0)  3 (50.0)  0 | 0.04* | 1. (ref)   2.37 (0.17-33.20)  3.78 (0.54-26.60)  0.33 (0.03-3.08) | 0.52  0.18  0.33 |
| **MELD (IQR)** | 8 (7-10) | 8 (6-10) | 0.37 |  |  |
| **Underlying cirrhosis (%)**  Yes  No | 40 (80.0)  5 (83.3) | 10 (20.0)  1 (16.7) | 1.0 |  |  |
| **BCLC Stage (%)**  A  B  C | 7 (70.0)  21 (84.0)  17 (81.0) | 3 (30.0)  4 (16.0)  4 (19.0) | 0.611 |  |  |
| **AFP (ng/mL, IQR)** | 13 (5-243) | 36.5 (6-207) | 0.88 |  |  |
| **Size of lesion (cm, IQR)** | 4.2 (2.7-8.1) | 4.2 (2.7-9.1) | 0.76 |  |  |
| **Location of lesion**  Multiple vs single segment(s) (%)  Post/Sup vs Ant/Inf (%)  Right vs left lobe (%) | 15 (83.3) vs 29 (78.4)  14 (87.5) vs 26 (76.5)  33 (80.5) vs 10 (76.9) | 3 (16.7) vs 8 (21.6)  2 (12.5) vs 8 (23.5)  8 (19.5) vs 3 (23.1) | 1.0  0.47  1.0 | 1.70 (0.25- 11.76) | 0.59 |
| **Distance of lesion from capsule (cm)** | 3.5 (1.5-5.5) | 2.5 (1.5-6.5) | 0.47 |  |  |
| **Operator (%)**  1  2  3  4  Other | 7 (87.5)  19 (73.1)  4 (100.0)  4 (80.0)  11 (84.6) | 1 (12.5)  7 (26.9)  0  1 (20.0)  2 (15.4) | 0.85 |  |  |
| **Imaging guidance (%)**  Ultrasound only  CT used | 41 (83.7)  4 (57.1) | 8 (16.3)  3 (43.9) | 0.10 | 1.0 (ref)  0.24 (0.3-1.78) | 0.16 |
| **Number of biopsies sent for pathology (IQR)** | 2 (2-3) | 2 (1-3) | 0.75 |  |  |

IQR: interquartile range; CI: confidence interval; OR: odds ratio; HCC: hepatocellular carcinoma; HCV: hepatitis C virus; NAFLD: non-alcoholic fatty liver disease; HBV: hepatitis B virus; MELD: model for end-stage liver disease; BCLC: Barcelona Clinic Liver Cancer; AFP: alpha-fetoprotein;

| **Supplemental Table 2. Risk Factors for Biopsy Complications** | | | |
| --- | --- | --- | --- |
|  | **Any Complication**  **(n=5)** | **No Complication**  **(n=52)** | **p-value** |
| **Age (SD)** | 60.6 (4.8) | 64.9 (8.2) | 0.25 |
| **Gender (%)**  Female  Male | 3 (33.3)  2 (4.2) | 6 (66.7)  46 (95.8) | 0.03* |
| **Race/Ethnicity (%)**  White  Black/African American  Other | 1 (2.4)  3 (23.1)  1 (20.0) | 41 (97.6)  10 (76.9)  4 (80.0) | 0.03* |
| **Etiology of HCC (%)**  HCV/alcohol  HCV  Alcohol  NAFLD  Other | 3 (21.4)  1 (5.0)  0  0  1 (8.3) | 11 (78.6)  19 (95.0)  8 (100.0)  6 (100.0)  11 (91.7) | 0.46 |
| **MELD (IQR)** | 7 (6-8) | 8 (7-10) | 0.32 |
| **Underlying cirrhosis (%)**  Yes  No | 4 (7.5)  1 (14.3) | 49 (92.5)  6 (85.7) | 0.48 |
| **BCLC Stage (%)**  A  B  C | 0  3 (11.5)  2 (8.7) | 11 (100.0)  23 (88.5)  21 (91.3) | 0.83 |
| **Platelets < 100,000 or**  **INR > 1.5 (%)**  Yes  No | 0  5 (9.6) | 7 (100.0)  47 (90.4) | 1.0 |
| **Size of lesion (cm IQR)** | 3.9 (2.1-4.8) | 4.2 (2.7-8.9) | 0.28 |
| **Location of lesion**  Multiple vs single segment (%)  Post/Sup vs Ant/Inf (%)  Right vs left lobe (%) | 1 (5.0) vs 4 (10.3)  2 (12.5) vs 3 (7.9)  4 (9.5) vs 1 (6.3) | 19 (95.0) vs 35 (89.7)  14 (87.5) vs 35 (92.1)  38 (90.5) vs 15 (93.8) | 0.65  0.63  1.0 |
| **Distance from capsule (cm)** | 1.5 (1.5-2.5) | 3.5 (2.0-5.5) | 0.16 |
| **Number of core biopsies (IQR)** | 9 (8-10) | 8 (7-9) | 0.34 |
| **Pathology (%)**  HCC  CCA or cHCC-CCA  No malignancy | 3 (7.5)  2 (40.0)  0 | 37 (92.5)  3 (60.0)  11 (100.0) | 0.07 |
| **Operator (%)**  1  2  3  4  Other | 0  2 (7.7)  0  0  3 (21.4) | 8 (100.0)  24 92.3)  7 (100.0)  5 (100.0)  11 (78.6) | 0.47 |
| **Gelfoam Used (%)**  Yes  No | 2 (4.8)  3 (16.7) | 40 (92.3)  15 (83.3) | 0.15 |
| **Imaging guidance (%)**  Ultrasound only  CT only | 4 (7.7)  1 (16.7) | 47 (92.3)  7 (87.5) | 0.47 |

SD: standard deviation; IQR: interquartile range; CI: confidence interval; HCC: hepatocellular carcinoma; HCV: hepatitis C virus; NAFLD: non-alcoholic fatty liver disease; HBV: hepatitis B virus; MELD: model for end-stage liver disease; BCLC: Barcelona Clinic Liver Cancer; AFP: alpha-fetoprotein; INR: international normalized ratio; CCA: cholangiocarcinoma; cHCC-CCA: combined hepatocellular cholangiocarcinoma
